# Stability analysis of slope based on the coupling of well-point dewatering and chemical improvement slope stabilization

**DOI:** 10.1371/journal.pone.0333430

**Published:** 2025-10-06

**Authors:** Shaoqiang Chai, Haidong Tu, Fengbo Dong, Deying Tang, Dingjie Tan, Qiumeng Yuan

**Affiliations:** 1 China Communications Construction First Highway Engineering Co., Ltd., Zhengzhou, Henan, China; 2 College of River and Ocean, Chongqing Jiaotong University, Chongqing, China; Tribhuvan University, NEPAL

## Abstract

This study investigates the impact of coupling well-point dewatering with chemical improvement slope stabilization configurations on slope stability. Targeting two slope stabilization types—toe-fixed pier and equidistant borehole configurations—the variation patterns of slope stability coefficients under different dewatering depths, pier parameters, and borehole parameters were analyzed using Geo Studio software. The results demonstrate that dewatering depth significantly enhances slope stability, with an optimal depth of 10 meters effectively reducing pore water pressure at the slip surface and improving stability. For the pier-type configuration, increasing pier height and width within a specific range notably enhances stability, though marginal benefits diminish gradually. In the borehole-type configuration, when the borehole depth is within the range of 2–4 meters, the reinforcement effect on shallow areas is particularly significant, and the slope stability coefficient can be increased by approximately 8.7%. Appropriately reducing the borehole spacing helps to further enhance stability; however, when the spacing is less than 1 meter, the improvement in slope stability is limited. These findings provide a theoretical basis and engineering guidance for optimizing slope stability designs under coastal complex geological conditions.

## Introduction

The stability of foundation pit slopes is an important issue in coastal area construction [1], especially in environments with high groundwater levels and complex geological conditions, where the risk of slope instability during excavation is significantly increased [2,3]. Due to the large difference in water head between the inside and outside of the foundation pit, the scouring effect of groundwater seepage on the slip surface has become a key factor affecting slope stability [4]. Implementing dewatering measures to reduce the foundation pit water level can not only reduce the water head difference and weaken the groundwater seepage capacity but also effectively enhance the overall stability of the slope [5]. However, the dewatering effect is significantly influenced by dewatering depth, soil permeability, and slope stabilization configurations [6,7], thus, it is of great importance to conduct in-depth research on the stability variation patterns under different factors.

Rainfall is one of the main external factors inducing slope instability [8]. The infiltration of rainwater raises the groundwater level, softens the soil of the potential slip surface, and leads to a decrease in the shear strength of the slip surface, thereby forming a landslide [9]. In recent years, scholars have carried out extensive research on the stability of slopes under rainfall conditions [10,11]. For example, Xiao Jiefu [12] revealed the deformation characteristics and instability mechanisms of ancient landslides on reservoir banks under reservoir water fluctuation and rainfall conditions through physical model tests. Wang [13] considered the effects of surface runoff and infiltration during rainfall and proposed a coupled method of rainfall hydrology and geomechanics to analyze the deformation and failure mechanisms of slopes under rainfall conditions. Liu Mingwei et al. [14] used steady-state and transient seepage field simulations to reveal the dynamic impact of rainfall on pore water pressure at the slip surface. In addition, some studies have focused on the rapid instability mechanisms of slopes under heavy rainfall conditions, such as the “J”-shaped change in the wetting line at the toe of the slope and the dynamic weakening of the slope’s shear strength [15]. As rainwater infiltrates and transfers within the slope, the physical and mechanical parameters of the rock and soil, the most dangerous sliding surface, and the safety factor all change accordingly. To address the above issues, the combination of dewatering and chemically improved slope stabilization techniques [16,17] has attracted attention as an effective means of enhancing slope stability [18]. The toe-fixed pier and equidistant borehole slope stabilization configurations are two common chemical improvement schemes, which can effectively restrain the sliding trend by increasing the shear strength of the soil at the slip surface [19]. However, the applicability and stability enhancement effects of different slope stabilization configurations under different dewatering depths and slope stabilization parameters have not been systematically studied, and there is a lack of quantitative research on their optimal design.

Therefore, in this paper, numerical simulations using the finite element method were conducted to investigate the coupling effect of well-point dewatering and chemical improvement slope stabilization techniques. The influence patterns of different dewatering depths, pier parameters, and borehole parameters on slope stability were systematically analyzed. The research results can provide a theoretical basis and engineering guidance for the design of foundation pit slope stability under complex geological conditions.

## Overview of foundation pit slope

This study is based on the Hangzhou section of the Class III waterway regulation project of the Beijing-Hangzhou Grand Canal in Zhejiang Province. The project area is located in a coastal sedimentary environment with strong tidal action, characterized by a high groundwater level and significant seepage characteristics of the foundation pit retaining structure. Geological surveys indicate that the foundation is mainly composed of silt, with particle components (particle size > 0.075 mm) accounting for less than 50% of the total weight. It has adverse engineering characteristics such as high water content, low shear strength, significant compressive deformation, and strong permeability. In addition, the physical and mechanical parameters of the soil in different sections show obvious spatial variability, and this geological heterogeneity poses a special challenge for the control of foundation pit stability.

**Fig 1:**
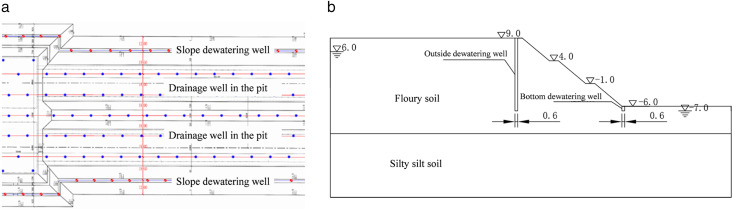
Schematic diagram of dewatering well arrangement. (a) Plan layout of dewatering wells. (b) Sectional layout of dewatering wells.

The foundation pit slope is adjacent to the Qiantang River. During construction, the strong permeability of the foundation pit and the high mobility of groundwater make the formation of a water head difference between the inside and outside of the foundation pit a significant threat to slope stability. To address the above issues, this study proposes a synchronous dewatering scheme: dewatering wells (referred to as “pit dewatering wells”) are arranged at the bottom of the foundation pit to maintain a dry construction environment within the pit; slope dewatering wells (referred to as “slope dewatering wells”) are arranged on the slope platforms of the stepped excavation. The dewatering method adopts open dewatering, which enhances slope stability by reducing the phreatic level. The plan and section layout of the dewatering wells are shown in Fig 1. (No permits were required for the described study, which complied with all relevant regulations.)

## Stability numerical simulation

### Establishment of slope finite element model

**Fig 2:**
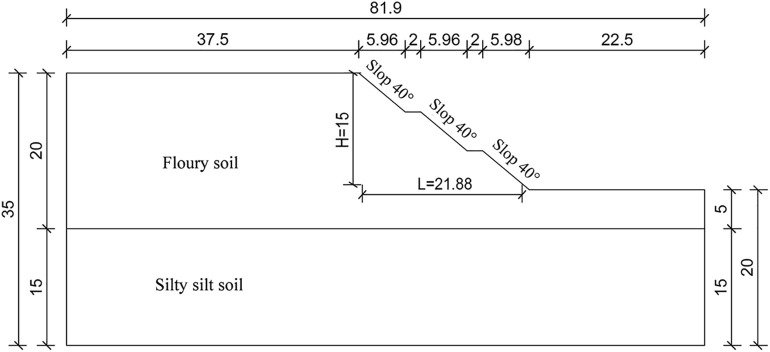
Schematic diagram of slope model.

To explore the stability variation patterns of different slope stabilization configurations improved by well-point dewatering, this paper selects water glass with a dosage of 6% as the soil improver and establishes two types of improved slope models: toe-fixed pier and equidistant borehole. The slope height and width are 15 m and 21.88 m, respectively, with a three-tiered stepped slope platform with a gradient of 1:1.2 and a 2-m-wide graded berm. According to the research results of Zheng Yiren [20] on the boundary effects of the strength reduction method, the slope model’s upper and lower boundaries should be more than twice the slope height, and the left and right boundaries should be 1.5 and 2.5 times the slope height, respectively, to achieve the most ideal calculation results. Therefore, this paper selects 35 m and 81.9 m as the upper and lower and left and right boundaries of the model, with the geometric shape shown in Fig 2.

**Fig 3:**
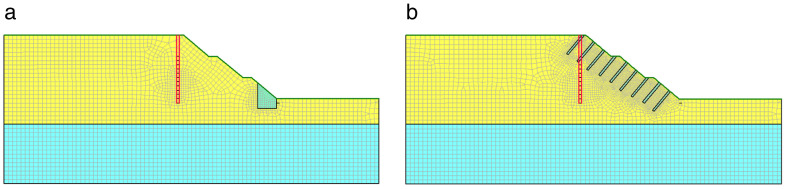
Numerical model of slope improved by well-point dewatering with different configurations. (a) Slope with fixed-pier stabilization at toe. (b) Slope with equidistant drilling stabilization.

The toe-fixed pier slope stabilization configuration sets pier-like improved soil at the toe of the slope, which enhances the shear strength of the soil at the slip surface to restrain sliding. The equidistant borehole slope stabilization configuration reinforces the slope by grouting through boreholes. The Geo Studio numerical models of the two configurations improved by well-point dewatering are shown in Fig 3.

### Boundary conditions and calculation parameters

(1) Boundary Conditions

The pressure at the bottom of the dewatering well in this paper is set to 0 to simulate the process of lowering the groundwater level in actual engineering. When groundwater exists in the strata around the dewatering well, due to the pressure head difference formed between the dewatering well and the foundation pit, water will move towards the dewatering well along the pressure gradient. This process continues until the water level in the strata around the foundation pit is lowered to the designed dewatering depth, the pore water pressure approaches zero, and the seepage process stops. This setting can more realistically reflect the dynamic process of foundation pit dewatering in actual engineering and ensure the consistency between the numerical simulation results and the actual situation. Based on the dewatering wells arranged before construction, this study assumes that the groundwater level at the bottom of the foundation pit is controlled 1 meter below the base and uses this as a constraint condition to focus on analyzing the impact of different dewatering depths of the slope-top dewatering wells on slope stability.

In terms of model boundary condition settings, the model’s side boundaries are defined as impermeable boundaries with restricted horizontal movement; the bottom is an impermeable boundary with restricted horizontal and vertical movements; the top, as the ground surface, is set as a seepage boundary without restrictions on horizontal and vertical movements. The groundwater levels on the left and right sides of the model are set as constant head boundaries to simulate the hydrological characteristics of the strata. In addition, this study uses a given head as the initial condition to establish the hydrological state of the slope before foundation pit dewatering, which serves as the basis for subsequent seepage and stability analyses.

(2) Selection of Calculation Parameters

The soil layers involved in this paper mainly include silt, silty clay, and silt improved with 6% water glass. The physical and mechanical parameters of each soil layer are shown in Table 1.

**Table 1:** Soil layer parameters.

Soil Layer	Density(kg/m^3^)	Elastic Modulus (Pa)	Poisson’s Ratio	Cohesion (kPa)	Friction Angle (°)
Silt	1940	1.1 × 10^7^	0.25	7.18	24.04
Improved Silt	2100	2.6 × 10^7^	0.23	24.10	25.13
Silty Clay	1790	2.5 × 10^6^	0.30	10.50	7.40

In addition to the soil layer parameters, the dewatering equivalent radius, dewatering depth, and total inflow of the foundation pit are also important calculation parameters. This project involves a rectangular foundation pit with a length of 100 meters and a width of 60 meters, which has a large excavation area and span. Therefore, a closed circular well-point system is used for foundation pit dewatering, with a dewatering depth of less than 20 m, set at 7–13 m in this paper.

By converting the rectangular foundation pit into a circular one, the equivalent radius of foundation pit dewatering is found to be 46.4 m. Combining Kuznetsov’s formula [21] and Dupuit’s formula [22], the pumping influence radius of the well-point system and the total inflow of the foundation pit under different dewatering depths are obtained, as shown in Table 2.

**Table 2:** Influence radius of well-point system pumping and total water inflow of foundation pit under different dewatering depths.

No.	Dewatering Depth (m)	Pumping Influence Radius (m)	Total Inflow of Foundation Pit (m^3^/d)
1	13	60.66	266.50
2	12	55.99	262.15
3	11	51.33	256.03
4	10	46.66	248.04
5	9	42.00	238.06
6	8	37.33	225.94
7	7	32.66	211.50
8	6	28.00	194.47

### Numerical calculation scheme design

This paper selects two types of improved slopes—slope with fixed-pier stabilization at toe and slope with equidistant drilling stabilization—for stability research after well-point dewatering. For the slope with fixed-pier stabilization at toe, the main influencing factors include pier width and pier height. For the slope with equidistant drilling stabilization, the borehole spacing and borehole depth are the main considerations. Therefore, to deeply analyze the stability of improved slopes under different factors, the following calculation conditions are set (as shown in Table 3).

**Table 3:** Calculation conditions with different slope stabilization types under different dewatering depths.

Slope Stabilization Type	Influencing Factor	Calculation Values				
Slope with Fixed-Pier Stabilization at Toe	Pier Width (m)	3				
	Pier Height (m)	0	0.5	1		
	Dewatering Depth (m)	7 ~ 13	7 ~ 13	7 ~ 13		
	Pier Height (m)	0.5				
	Pier Width (m)	2	3	4	5	
	Dewatering Depth (m)	7 ~ 13	7 ~ 13	7 ~ 13	7 ~ 13	
Slope with Equidistant Drilling Stabilization	Borehole Spacing (m)	1				
	Borehole Depth (m)	2	3	4	5	6
	Dewatering Depth (m)	7 ~ 13	7 ~ 13	7 ~ 13	7 ~ 13	7 ~ 13
	Borehole Depth (m)	5				
	Borehole Spacing (m)	2.5	2	1.5	1	0.5
	Dewatering Depth (m)	7 ~ 13	7 ~ 13	7 ~ 13	7 ~ 13	7 ~ 13

## Numerical simulation results and discussion

### Seepage field and pore pressure distribution characteristics

**Fig 4:**
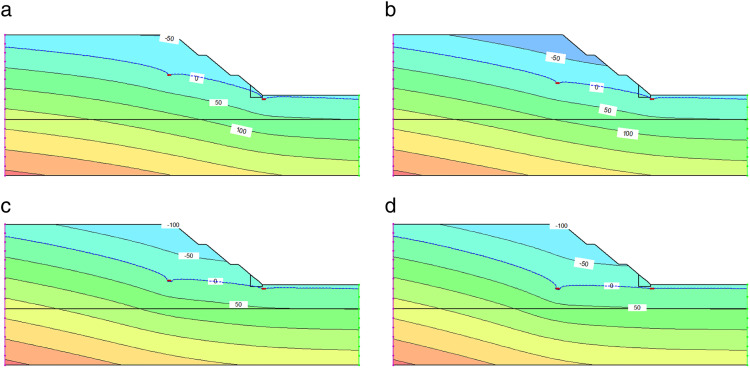
Pore water pressure distribution nephogram of slope improved by fixed-pier stabilization at toe under different dewatering depths. (a) Dewatering depth of 7 m. (b) Dewatering depth of 9 m. (c) Dewatering depth of 11 m. (d) Dewatering depth of 13 m.

Taking the slope improved by fixed-pier stabilization at toe with a pier height of 0.5 m and a pier width of 3 m as a typical case, the variation patterns of pore water pressure under different dewatering depths are analyzed. It is found that the well-point dewatering process of the foundation pit slope is a dynamic process, with specific changes as shown in Fig 4.

It can be seen from Fig 4 that before dewatering begins, the water surface within the foundation pit slope maintains the natural water level, and the pore water pressure increases linearly with depth. After dewatering starts, groundwater near the dewatering well moves towards the pumping well due to the pressure gradient, gradually forming a conical dewatering curve, known as the “dewatering cone.” The curvature of the dewatering cone is small at the initial moment and gradually deepens over time, forming a large curvature “cone bottom” near the well bottom. In addition, the pore water pressure distribution nephogram shows that the pore water pressure at the bottom of the dewatering well is zero, and the pressure increases gradually with the distance from the dewatering well. The groundwater level line is located at the intersection of positive and negative pore water pressures, where the pressure is zero. The area below the water level line has positive pore water pressure, which increases with depth, while the area above the water level line is a negative pressure zone, where the influence of pore water pressure on soil shear strength is significantly reduced.

**Fig 5:**
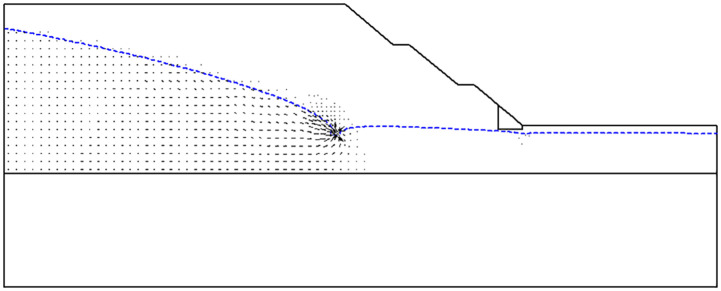
Velocity vector diagram of slope improved by fixed-pier stabilization at toe under dewatering depth of 13 m.

Further analysis of the velocity vector diagram at the bottom of the dewatering well of the slope improved by fixed-pier stabilization at toe under a dewatering depth of 13 m (as shown in Fig 5) reveals that the seepage velocity at the well bottom is the highest. This is because the largest pressure gradient is formed between the bottom of the dewatering well and the strata, resulting in a significant increase in the water flow rate. The concentrated change in seepage velocity has a significant impact on the strength of the soil around the well bottom, which may in turn affect the long-term stability of well-point dewatering.

### Slope slip surface and stability analysis

**Fig 6:**
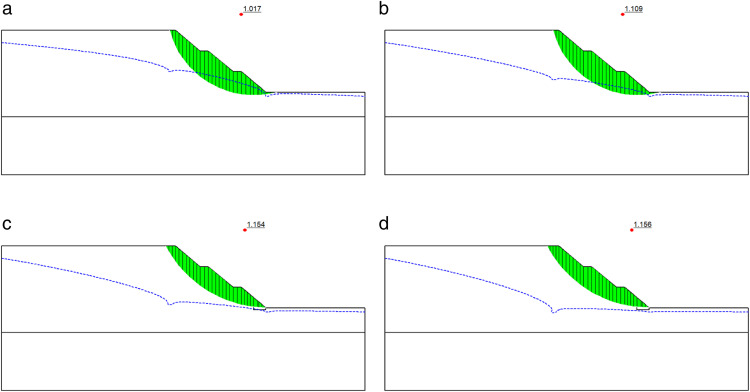
Slip surfaces of slope improved by fixed-pier stabilization at toe under different dewatering depths. (a) Dewatering depth of 7 m. (b) Dewatering depth of 9 m. (c) Dewatering depth of 11 m. (d) Dewatering depth of 13 m.

The impact of different well-point dewatering depths on the slip surface is of great importance, as it directly relates to the stability of the improved slope. To analyze the specific variation patterns of the slip surface, the slope improved by fixed-pier stabilization at toe with a pier height of 0.5 m and a pier width of 3 m is still taken as a typical case for calculation, and the results are shown in Fig 6.

As illustrated in Fig 6, as the dewatering depth increases from 7 m to 13 m, the pore water pressure near the slip surface gradually decreases, accompanied by a continuous decline in the water table. The zone of soil below the water table consequently expands, leading to enhanced shear strength of the soil adjacent to the slip surface. Consequently, the overall slope stability exhibits an upward trend. Specifically, at a dewatering depth of 7 m (Fig 6(a)), the main body of the slip surface remains above the water table. The soil maintains a high moisture content with elevated saturated unit weight, resulting in an increased downslide moment and consequently lower stability. When the dewatering depth reaches 9 m (Fig 6(b)), the water table descends further, initiating a reduction in moisture content within the slip surface zone. The diminished downslide moment contributes to an improvement in the stability factor. At 11 m dewatering depth (Fig 6(c)), the water table drops below the slip surface, causing a significant reduction in pore water pressure. The soil near the slip surface transitions away from saturation, reducing its unit weight while further enhancing shear strength, thereby markedly increasing the stability factor. With the dewatering depth extended to 13 m (Fig 6(d)), the slip surface lies entirely within the negative pore pressure zone, where pore water pressure approaches zero. The stability factor progressively approaches saturation, and further increases in dewatering depth yield diminishing returns on stability enhancement.

**Fig 7:**
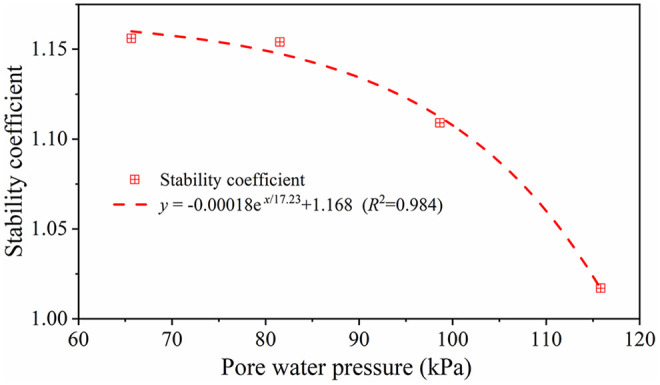
Variation relationship between pore water pressure and stability coefficient.

By extracting pore water pressure at fixed locations of the slope and corresponding stability coefficients under varying dewatering depths, a regression model was established to characterize their relationship, with results presented in Fig 7(S1 Table provides the original data of Fig 7). Statistical analysis reveals a significant negative correlation between pore water pressure and stability coefficient (*R*² = 0.984). Mechanistically, reduced pore water pressure decreases soil water content, which concurrently lowers unit weight and increases effective stress, ultimately enhancing shear strength. This demonstrates that well-point dewatering actively regulates pore water pressure to modify soil physico-mechanical states, essentially augmenting effective stress in the soil skeleton to improve anti-sliding stability.

**Fig 8:**
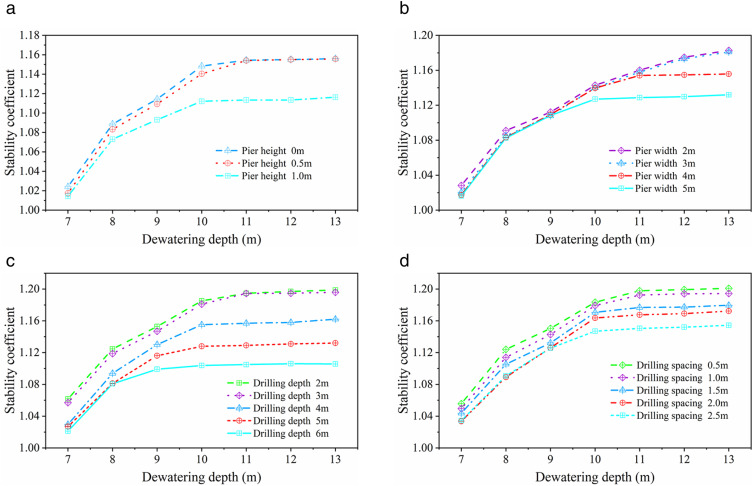
Variation patterns of stability coefficient for improved slope under different influencing factors. **(A)** Effect of different pier heights. **(B)** Effect of different pier widths. **(C)** Effect of different borehole depths. **(D)** Effect of different borehole spacings.

To further analyze the impact of different influencing factors on the stability of the improved slope, stability calculations were performed using the SLOPE/W module in Geo Studio software based on the conditions listed in Table 3, and the results are shown in Fig 8. (S2 Table provides the original data of Fig 8 (a–d))

Fig 8 shows the stability variation curves of the two types of improved slopes (slope with fixed-pier stabilization at toe and slope with equidistant drilling stabilization) under different well-point dewatering depths. For the slope with fixed-pier chemical improvement at toe, when the pier height increases from 0 to 0.5 m, the stability factor significantly increases by approximately 3.6%, indicating a noticeable restraining effect of the pier height on the slip surface. However, when the pier height is further increased to 1 m, the change in the stability factor is less than 0.1%, indicating that the effect of pier height on slope stability enhancement tends to saturate (see Fig 8(a)). For footing piers with low height-to-diameter ratios, excessive pier height does not significantly enhance flexural and anti-overturning capacities. Lateral loads are primarily resisted by the upper-middle section of the pier and shallow front soil layers, resulting in suboptimal mobilization of deep soil resistance. This phenomenon aligns mechanically with the conclusion in Reference [23]: “Reduced embedment depth diminishes load transfer efficiency to deep soil strata.” Consequently, height escalation yields diminishing returns in sliding resistance improvement, while potentially inducing material waste and compromised cost-effectiveness. In addition, when the pier width increases from 2 m to 4 m, the stability factor significantly improves, but when the pier width exceeds 5 m, the increase is less than 0.2%, indicating that the marginal benefit of increasing pier width gradually decreases (see Fig 8(b)).

For the slope with equidistant drilling chemical improvement, when the borehole depth is between 2 and 4 m, the stability factor significantly increases with the increase in borehole depth, by approximately 8.7%. At this time, the boreholes mainly act on the shallow area of the slip surface, improving the mechanical properties of the surface soil of the slope (see Fig 8(c)). When the borehole depth is larger, and the boreholes exceed the main control zone of the slip surface, the increase in the stability factor gradually decreases, showing a diminishing marginal benefit. For deep boreholes above 6 m, the further increase in borehole depth has limited impact on stability enhancement, with an increase of less than 0.5%. The borehole spacing has a more sensitive impact on stability. When the spacing decreases from 2.5 m to 1 m, the stability factor significantly improves, but when the spacing is less than 1 m, the marginal benefit of further reduction is low (see Fig 8(d)).

### Factor sensitivity analysis

To quantify the influence of key parameters on slope stability in two stabilization methods, this study employs Range Analysis Method based on numerical simulation results illustrated in Fig 8(a)-(d). This approach evaluates parameter sensitivity by comparing the maximum difference (*R*) in the average stability coefficient (*K*) across varying factor levels. A larger *R* value indicates higher sensitivity, signifying greater impact on slope stability. Specifically, for slope with fixed-pier stabilization at toe, we first compute the mean stability coefficient *K* at each factor level: when pier width = 2 m, derive *K*_1_ by averaging stability coefficients across all dewatering depths; subsequently calculate *K*_2_, *K*_3_, and *K*_4_ for widths of 3 m, 4 m, and 5 m respectively, with identical procedures applied to pier height and dewatering depth variations. The range *R* for each factor is then determined via Equation (1), directly correlating the magnitude of *R* to parametric influence intensity.


$$R=\max\left\{K_{1},K_{2},...,K_{i}\right\}-\min\left\{K_{1},K_{2},...,K_{i}\right\}$$ (1)

In Equation (1), *R* denotes the range of each influencing factor, *K*_i_ represents the mean stability coefficient of the slope at the i-th level of the factor.

For slope with fixed-pier stabilization at toe, the computed ranges (*R*) of pier width, pier height, and dewatering depth are 0.024, 0.029, and 0.142, respectively. Range comparisons reveal that dewatering depth exerts the most pronounced influence on slope stability, followed by pier height, while pier width demonstrates the least impact. Correspondingly, for slope with equidistant drilling stabilization, the ranges for borehole depth, borehole spacing, and dewatering depth are 0.071, 0.035, and 0.130. Results consistently indicate that dewatering depth remains the dominant controlling factor, with borehole depth exhibiting secondary influence and borehole spacing proving least influential.

As comprehensively demonstrated, well-point dewatering depth emerges as the parameter with the highest sensitivity across all variables, underscoring its primacy in slope stability control within coastal high-water-table zones where effective groundwater management is critical. Furthermore, strategic coordination of structural parameters among different stabilization methods—such as optimizing pier-to-borehole geometric ratios or coupling dewatering with reinforcement density—contributes synergistically to enhanced stability margins, translating theoretical sensitivity insights into practical design efficacy.

### Economic analysis of different slope stabilization methods

To achieve optimal balance between slope stability and construction cost-effectiveness, an economic analysis is conducted based on prior computational results, adhering to specifications requiring a minimum stability coefficient of 1.05 for Grade IV slopes during construction. For slope with fixed-pier stabilization at toe, two configurations are compared: (a) pier height H = 0.5 m with width B = 2 m, and (b) H = 0 m with B = 3 m. For slope with equidistant drilling stabilization, four configurations are assessed: (a) borehole spacing S = 1 m with depth D = 5 m, (b) S = 1.5 m, D = 5 m, (c) S = 1 m, D = 2 m, and (d) S = 2.5 m, D = 5 m.

**Fig 9:**
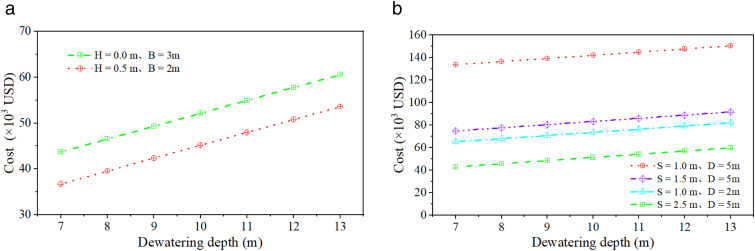
Total cost vs. dewatering depth. **(a)** Slope with fixed-pier stabilization at toe. **(b)** Slope with equidistant drilling stabilization. (S3 Table provides the original data of Fig 9).

As indicated in Fig 9(a), the total cost of fixed-pier stabilization exhibits a linear increase with deeper well dewatering depths. Therefore, while ensuring compliance with stability requirements, the dewatering depth should be rationally determined based on site-specific conditions to minimize pumping costs and enhance economic efficiency. For a fixed dewatering depth, the configuration with pier height H = 0.5 m and width B = 2 m proves more economical than the alternative with H = 0 m and B = 3 m. Specifically, the minimal total cost is achieved under the optimal configuration: dewatering depth = 7 m, H = 0.5 m, and B = 2 m.

Fig 9(b) demonstrates that the total cost of equidistant drilling stabilization likewise exhibits linear growth with increasing dewatering depth, consistent with the trend observed for slope-toe footing piers. Overall, the configuration with borehole spacing S = 1 m and depth D = 5 m incurs the highest total cost, while other borehole schemes show comparable expenditure levels. For a given dewatering depth, the combination of spacing S = 2.5 m and depth D = 5 m achieves the lowest total cost.

## Conclusions

Based on numerical simulations using Geo Studio, this paper systematically studied the coupling effect of well-point dewatering and two slope stabilization configurations (slope with fixed-pier stabilization at toe and slope with equidistant drilling stabilization) on the stability of improved slopes, and analyzed the influence patterns of dewatering depth, pier parameters, and borehole parameters on the stability factor. The main conclusions are as follows:

1. Within the dewatering depth range of 7–10 m, the pore water pressure at the slip surface significantly decreases, and the slope stability factor shows a noticeable increase. However, when the dewatering depth exceeds 10 m, the water level line completely drops below the slip surface, and the change in the stability factor tends to flatten, with limited marginal benefits from further increasing the dewatering depth.
2. For the slope with fixed-pier chemical improvement at toe, increasing the pier height to 0.5 m significantly improves the stability factor by approximately 3.6%. However, further increasing the height to 1 m leads to a saturated stability factor. Additionally, increasing the pier width to 3 m significantly enhances the stability factor, but beyond 5 m, the increase is limited.
3. For the slope with equidistant drilling chemical improvement, the reinforcement effect on the shallow area of the slip surface is most significant when the borehole depth is between 2 and 4 m, with an increase in the stability factor of approximately 8.7%. However, when the borehole depth exceeds 5 m, the reinforcement effect on the deep area of the slip surface tends to saturate, with an increase of less than 0.5%. The borehole spacing has a more sensitive impact on stability. When the spacing decreases from 2.5 m to 1 m, the stability factor significantly improves, but when the spacing is less than 1 m, the marginal benefit of further reduction is low.

This study employed numerical simulation methods to systematically analyze slope stability under static dewatering conditions. However, in practical engineering, especially during construction in rainy seasons, the safety margin of slopes may decrease due to rainfall infiltration. Future research could build on this work by incorporating transient seepage analysis and combining experimental and simulation approaches to further investigate the influence mechanisms of the combined effects of rainfall and well-point dewatering on slope stability. This would provide a more comprehensive and reliable basis for the design of related engineering projects.

## Supporting information

S1 Table The original data in Fig 7. (PDF)

S2 Table The original data in Fig 8. (PDF)

S3 Table The original data in Fig 9. (PDF)
