# Supplementary material for: Stability analysis of slope based on the coupling of well-point dewatering and chemical improvement slope stabilization
Source: PLoS One. 2025 Oct 6;20(10):e0333430. doi: 10.1371/journal.pone.0333430 (PMC12500126; doi:10.1371/journal.pone.0333430)
Supplement: S1 Table — (PDF) [file pone.0333430.s001.pdf]

S1 Table. The original data in Fig 7

pore water pressure (kPa)	Stability coefficient
65.658	1.156
81.534	1.154
98.646	1.109
115.833	1.017
