# Supplementary material for: Stability analysis of slope based on the coupling of well-point dewatering and chemical improvement slope stabilization
Source: PLoS One. 2025 Oct 6;20(10):e0333430. doi: 10.1371/journal.pone.0333430 (PMC12500126; doi:10.1371/journal.pone.0333430)
Supplement: S2 Table — (PDF) [file pone.0333430.s002.pdf]

S2 Table (a). The original data in Fig 8(a)

Dewatering depth (m)	Stability coefficient		
	Pier height 0m	Pier height 0.5m	Pier height 1m
7	1.02407	1.01743	1.01428
8	1.08862	1.08322	1.07302
9	1.11436	1.10923	1.09307
10	1.14831	1.14037	1.1121
11	1.15441	1.15406	1.1134
12	1.15502	1.15502	1.1134
13	1.15598	1.15564	1.11628

S2 Table (b). The original data in Fig 8(b)

Dewatering depth (m)	Stability coefficient			
	Pier width 2m	Pier width 3m	Pier width 4m	Pier width 5m
7	1.02824	1.02049	1.0173	1.01624
8	1.09099	1.08499	1.08324	1.08255
9	1.11218	1.10793	1.10937	1.10831
10	1.14287	1.13998	1.13968	1.12699
11	1.16012	1.15799	1.15411	1.12874
12	1.17493	1.17318	1.1548	1.1298
13	1.18268	1.18093	1.15586	1.13193

S2 Table (c). The original data in Fig 8(c)

Dewatering depth (m)	Stability coefficient				
	Borehole depth 2m	Borehole depth 3m	Borehole depth 4m	Borehole depth 5m	Borehole depth 6m
7	1.06183	1.05704	1.02961	1.02697	1.02106
8	1.1246	1.11868	1.09382	1.08118	1.08078
9	1.15283	1.14683	1.13012	1.11605	1.09901
10	1.18513	1.18097	1.15507	1.12796	1.10381
11	1.19472	1.1944	1.1569	1.12908	1.10493
12	1.19696	1.19472	1.15802	1.13092	1.10605
13	1.1988	1.19584	1.1621	1.13204	1.10565

S2 Table (d). The original data in Fig 8(d)

	Stability coefficient				
Dewatering depth (m)	Borehole spacing 0.5m	Borehole spacing 1m	Borehole spacing 1.5m	Borehole spacing 2m	Borehole spacing 2.5m
7	1.05575	1.04956	1.04371	1.03359	1.03442
8	1.12407	1.11395	1.10542	1.08912	1.09071
9	1.1505	1.14272	1.13219	1.126	1.12566
10	1.18345	1.17919	1.17066	1.16363	1.14699
11	1.19783	1.1924	1.17684	1.16756	1.1505
12	1.19934	1.19399	1.17726	1.16907	1.15201
13	1.20093	1.19432	1.1796	1.17224	1.15435
