# Supplementary material for: Stability analysis of slope based on the coupling of well-point dewatering and chemical improvement slope stabilization
Source: PLoS One. 2025 Oct 6;20(10):e0333430. doi: 10.1371/journal.pone.0333430 (PMC12500126; doi:10.1371/journal.pone.0333430)
Supplement: S3 Table — (PDF) [file pone.0333430.s003.pdf]

S3 Table (a). The original data in Fig 9(a)

Dewatering depth (m)	Cost (×10^3 USD)	
	H = 0.0 m、B = 3m	H = 0.5 m、B = 2m
7	43.714	36.725
8	46.524	39.535
9	49.334	42.345
10	52.144	45.155
11	54.953	47.964
12	57.763	50.774
13	60.573	53.586

S3 Table (b). The original data in Fig 9(b)

Dewatering depth (m)	Cost (×103 USD)			
	S = 1.0 m、D = 2m	S = 1.0 m、D = 5m	S = 2.5 m、D = 5m	S = 1.5 m、D = 5m
7	65.228	133.567	42.871	74.848
8	68.039	136.377	45.680	77.658
9	70.849	139.187	48.490	80.467
10	73.659	141.996	51.300	83.277
11	76.469	144.808	54.112	86.087
12	79.279	147.618	56.921	88.897
13	82.089	150.427	59.731	91.708
